# Speciation analysis of organoarsenic species in marine samples: method optimization using fractional factorial design and method validation

**DOI:** 10.1007/s00216-021-03341-4

**Published:** 2021-05-15

**Authors:** Jojo Tibon, Marta Silva, Jens J. Sloth, Heidi Amlund, Veronika Sele

**Affiliations:** 1 Institute of Marine Research, P.O. Box 1870, 5817 Bergen, Norway; 2 National Food Institute, Technical University of Denmark, Kemitorvet, Building 201, 2800 Kgs. Lyngby, Denmark

**Keywords:** Arsenic speciation, HPLC, ICP-MS, Marine certified reference materials

## Abstract

Organoarsenic species in marine matrices have been studied for many years but knowledge gaps still exist. Most literature focuses on monitoring of arsenic (As) species using previously published methods based on anion- and cation-exchange high-performance liquid chromatography-inductively coupled plasma mass spectrometry (HPLC-ICP-MS). These studies are often limited to few As species and/or only specific method performance characteristics are described. Most marine certified reference materials (CRMs) are only certified for arsenobetaine (AB) and dimethylarsinate (DMA), making it difficult to evaluate the accuracy of analytical methods for other organoarsenic species. To address these gaps, the main objective of this work was to develop and validate a method for speciation analysis of a broad range of organoarsenic species in marine matrices. Optimum extraction conditions were identified through a 2^7–3^ fractional factorial design using blue mussel as test sample. The effects of sample weight, type and volume of extraction solution, addition of H_2_O_2_ to the extraction solution, extraction time and temperature, and use of ultrasonication were investigated. The highest As recoveries were obtained by using 0.2 g as sample weight, 5 mL of aqueous methanol (MeOH:H_2_O, 50% v/v) as extractant, extraction carried out at 90 °C for 30 min, and without ultrasonication. Anion- and cation-exchange HPLC-ICP-MS settings were subsequently optimized. The method detected a total of 33 known and unknown As species within a run time of 23 and 20 min for cation-exchange and anion-exchange, respectively. A single-laboratory validation was conducted using several marine CRMs: BCR 627 (tuna fish tissue), ERM-CE278k (mussel tissue), DORM-4 (fish protein), DOLT-5 (dogfish liver), SQID-1 (cuttlefish), TORT-3 (lobster hepatopancreas), and CRM 7405-b (hijiki seaweed). Method performance characteristics were evaluated based on selectivity, limits of detection and quantification, linearity, trueness, precision, and measurement uncertainty. This work proposes an extraction procedure which allowed satisfactory quantification of As species with low solvent and energy consumption, supporting “Green Chemistry” principles. The study also presents a new set of As speciation data, including methylated arsenic species and arsenosugars, in recently issued marine CRMs, which will be valuable for future speciation studies on As. This work is the first to report a total of 33 different As species in marine CRMs.

**Supplementary Information:**

The online version contains supplementary material available at 10.1007/s00216-021-03341-4.

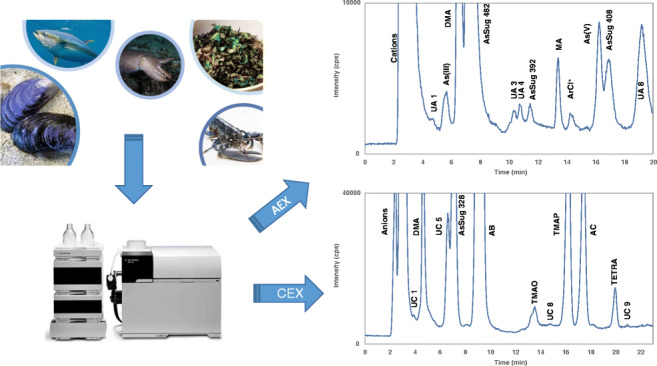

## Introduction

Marine organisms are known to accumulate arsenic (As) from their environment. The cycle usually starts with inorganic As (iAs) present in seawater, which is taken up by phytoplanktons and other organisms at lower trophic levels. These primary producers and consumers are preyed on by other marine animals, causing As to be transformed to organoarsenic species and biomagnified through the food chain [1]. Most monitoring studies report high total As concentrations in marine food products (8–22 mg/kg w.w.) [2], but only a small fraction (<1% of total As) exists as the toxic iAs [3] (sum of arsenite [As(III)] and arsenate [As(V)]). Some exceptions include hijiki (*Hizikia fusiforme*), a family of brown seaweed reported to have As(V) concentrations as high as 107 mg/kg d.w. [4], and blue mussels harvested from Norwegian fjords with unusual elevated levels of iAs (up to 5.8 mg/kg w.w.) [5]. Arsenobetaine (AB) is the predominant organoarsenic species found in most finfish and shellfish, typically accounting for more than 90% of the total As [6]. Seaweed is known to contain several arsenosugars (AsSug), as described in the analysis of edible algae samples [7]. Arsenolipids are prevalent in marine oils and fats [8] but were also reported in commonly consumed types of seafood [9]. Other methylated As species exist as minor components, with dimethylarsinate (DMA) being the most common [10]. Tetramethyl arsonium ion (TETRA) was observed to be the predominant species in some mollusks [11], while elevated levels of trimethylarsoniopropionate (TMAP) were found in crabs [2]. Trimethylarsine oxide (TMAO), methylarsonate (MA), and arsenocholine (AC) were observed in trace concentrations in most seafood [10]. The chemical structures of the most common As species can be found in an article by Luvonga et al. [11].

Based on the classification by the International Agency for Research on Cancer (IARC), iAs is a carcinogen, AB is generally regarded as non-toxic, while other methylated As species such as DMA and MA were classified as possibly carcinogenic [11, 12]. There are also discussions on the potential toxicity of arsenosugars and arsenolipids, with studies citing neurotoxic and cytotoxic effects [13–15]. The metabolism of these complex As species commonly found in seafood leads to formation of toxic dimethylated forms [16]. Considering the potential toxicity of the different organoarsenic species, it may not be sufficient to base the risk assessment on iAs alone. Hence, the European Food Safety Authority Panel on Contaminants in the Food Chain (CONTAM) emphasized the importance of As speciation data in different foodstuffs for a holistic evaluation of As exposure due to diet [17]. The recent findings highlight the need for robust, validated analytical methods for As speciation to contribute to the crafting of future food legislations, and subsequent routine monitoring and food control analysis. While European standard methods for iAs already exist [18, 19], a standardized method for organoarsenic species is still not issued.

In speciation analysis, mild extraction conditions are typically employed to liberate the analytes from the matrix while preventing conversion of species [1, 10, 20]. For the analysis of water-soluble As species, commonly used extraction solvents include pure water [21, 22], mixtures of methanol and water [23, 24], and mildly acidic solutions, e.g., nitric acid [25, 26]. An agitation and/or heating device is used to facilitate the extraction, e.g., a mechanical shaker/vortex mixer [23, 24], hotblock [21, 27], water bath [28], ultrasonic bath/probes [29], or microwave systems [22, 26]. By far, high-performance liquid chromatography (HPLC) using cation- and/or anion-exchange columns is still the most utilized technique in As speciation analysis. Inductively coupled plasma mass spectrometry (ICP-MS) is widely used as an arsenic-specific detection system due to its high sensitivity, good selectivity, and compatibility with separation instruments, especially HPLC [30].

Due to the distinct polarities of As species and complexities of the different matrices, a universal extraction procedure for all As species in all foodstuffs has not yet been developed. Thus, a targeted sample treatment has been recommended wherein extraction conditions are optimized specifically for the matrices and analytes of interest [20, 31]. Most method development studies are carried out using a univariate (“one-factor-at-a-time”) strategy, but this approach is rather time-consuming and laborious. A recommended alternative approach is to use multivariate optimization wherein variables are changed simultaneously, thereby allowing maximum gain of information with as few experiments as possible [32]. The use of design of experiments (DoE), such as a two-level factorial design, is commonly used for evaluation of factors with significant effects and interactions [33]. If dealing with several factors and if resources are constrained, a more pragmatic approach is a fractional factorial design. The DoE as a chemometric tool for method optimization has previously been used in speciation analysis of arsenic [34, 35], zinc [36], selenium [37], chromium [38], and mercury [39] in a wide range of matrices.

In a recent review by Ardini et al. [1] covering literature on As speciation analysis of environmental samples published from 2004 to 2018, almost half of the papers were devoted to investigation in marine organisms. Only around 25% delved into method optimization. In addition, out of the 200 papers reviewed, only 60% used CRMs, and only a third utilized CRMs in their method validation. To bridge this gap, the aims of the present study were (1) to perform extraction optimization using fractional factorial design with blue mussel as the test matrix, (2) to optimize HPLC-ICP-MS conditions, (3) to perform a single-laboratory validation using several marine matrices, and (4) to apply the method to a range of marine CRMs with an overall goal of providing information values which can be used as reference for evaluation or comparison of future analytical methods.

## Materials and methods

### Reagents and standards

All reagents used were analytical grade and of high purity. Methanol (MeOH, ≥ 99.97%), pyridine (C_5_H_5_N, ≥ 99.5%), formic acid (HCOOH, ≥ 98%), nitric acid (HNO_3_, 65%), hydrogen peroxide (H_2_O_2_, 30%), ammonia solution (NH_3_, 25%), and ammonium carbonate ((NH4)_2_CO_3_, reagent grade) were purchased from Merck (Darmstadt, Germany). Nitric acid was further purified using a sub-boiling distillation unit (Savillex, Eden Prairie, MN, USA). Acetonitrile (CH_3_CN/ACN, ≥ 99.95%) was obtained from VWR Chemicals BDH (Fontenay-sous-Bois, France). Ultrapure water (18.2 MΩ-cm) was produced in-house using a Milli-Q water purification system (Merck Millipore, Burlington, MA, USA) and was used throughout the study.

Arsenite [As(III)] and arsenate [As(V)] solutions (1000 mg/L) were produced by Spectrascan Teknolab (Ski, Norway). Arsenobetaine (AB, ≥ 95%) and a sodium salt of dimethylarsinic acid (DMA, ≥ 98%) were purchased from Sigma-Aldrich (St. Louis, MO, USA). Tetramethyl arsonium iodide (TETRA, 97%) and trimethylarsine oxide (TMAO, 95%) were supplied by Toronto Research Chemicals (Toronto, Ontario, Canada). The standard solution of arsenocholine (AC, 19.77 mg/kg) was produced by the National Institute of Standards and Technology (NIST, Gaithersburg, MD, USA), while monomethylarsonic acid (MA, 99.5%) was sourced from Chem Service, Inc. (West Chester, PA, USA). Standard solutions of other methylated arsenic species such as trimethylarsoniopropionate (TMAP), dimethylarsinoyl acetate (DMAA), dimethylarsinoyl ethanol (DMAE), and dimethylarsinoyl propionate (DMAP), as well as the glycerol-arsinoylriboside (AsSug 328) and other arsenosugars (AsSug 392, 408, and 482), were procured from the University of Graz (Austria). Stock solutions were prepared by dissolving or diluting appropriate amounts of the standards in water. Accurate As concentrations were determined by ICP-MS.

### Samples and reference materials

Blue mussel samples (*n* = 50) from the Norwegian surveillance programme for mussels in 2017 [40], led by the Norwegian Food Safety Authority, were pooled and homogenized using a food processor (Braun Multiquick 7 K3000, Kronberg im Taunus, Germany). These were subsequently freeze-dried for 72 h (Labconco FreeZone 18 L, Kansas City, MO, USA) and homogenized using a knife mill (Retch Grindomix GM 100, Haan, Germany). The resulting pooled sample served as an in-house quality control (QC) material and was analyzed for total As with 10 replicates. The average result was set as the target total As concentration. The blue mussel sample was used as test matrix for the extraction optimization using fractional factorial design. Blue mussel was chosen since previous studies reported the presence of several As species, including four to six unknowns [41, 42].

The certified reference materials (CRMs) utilized were tuna fish tissue (BCR 627), mussel tissue (*Mytilus edulis*, ERM-CE278k), and bladderwrack seaweed (*Fucus vesiculosus*, ERM-CD200) from the Institute for Reference Materials and Measurements of the European Commission’s Joint Research Centre (IRMM, Geel, Belgium); fish protein (DORM-3 and DORM-4), dogfish liver (*Squalus acanthias*, DOLT-5), cuttlefish (*Sepia pharaonis*, SQID-1), and lobster hepatopancreas (TORT-3) from the National Research Council Canada (NRC, Ottawa, Ontario, Canada); hijiki seaweed (*Hizikia fusiforme*, CRM 7405-b) from the National Metrology Institute of Japan (NMIJ, Ibaraki, Japan); and oyster tissue (*Crassostrea virginica*, SRM 1566b) from NIST (Gaithersburg, MD, USA).

### Experimental overview

**Fig. 1:**
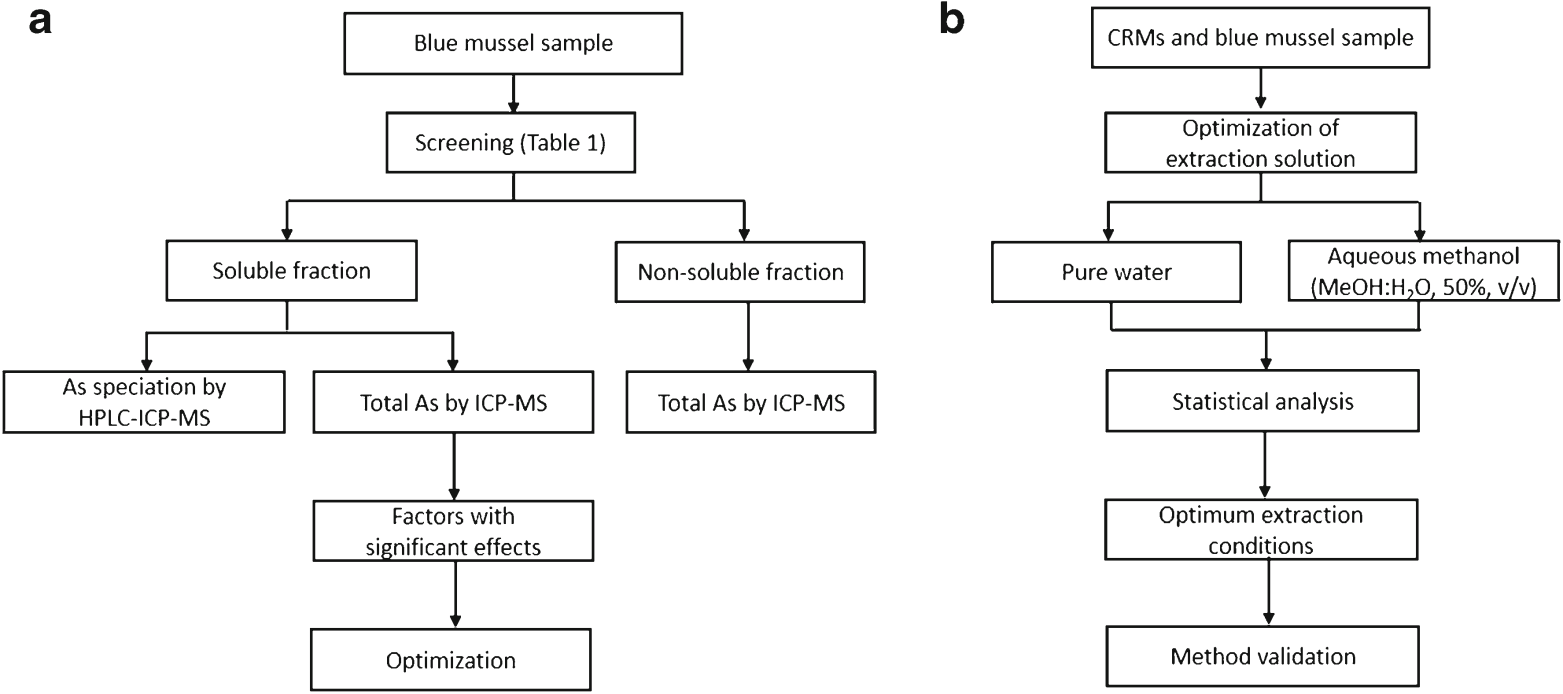
A process flow chart of the (a) screening and (b) optimization experiments leading to method validation using blue mussel and CRMs

In all experiments from the initial method development phase until validation, extraction efficiencies were evaluated by comparing the total As in the extracts and in the samples. Chromatographic recovery was assessed by comparing the sum of As species from HPLC-ICP-MS with the total As in the soluble extracts. Overall mass balance was checked to ensure that As in the different fractions were accounted for. A process flow chart summarizing the experiments performed in this study is presented in Fig. 1.

### Extraction optimization: screening of factors using fractional factorial design

Based on a review of extraction procedures used for As speciation in marine matrices [21, 26, 27], a total of seven factors were identified as the most important and were chosen for the experimental design: (A) sample weight (g), (B) type of extraction solution, (C) volume of extraction solution (mL), (D) addition of H_2_O_2_ in the extraction solution, (E) extraction temperature (°C), (F) extraction time (min), and (G) use of ultrasonication. A 2^7–3^ fractional factorial design was devised (resolution IV), with a total of 16 experiments performed in random order as described in Table 1. Total As concentrations in the soluble extracts were chosen as the response to optimize.

**Table 1:** 2^7 − 3^ fractional factorial design (resolution IV). The tested factors were (A) sample weight (g), (B) type of extraction solution, (C) volume of extraction solution (mL), (D) addition of H_2_O_2_, (E) extraction temperature (°C), (F) extraction time (min), and (G) use of ultrasonication. Coded factor levels are denoted as “−1” or “+1” followed by the real factor setting in parenthesis. Total arsenic concentration in the blue mussel sample was 14.6 ± 0.1 mg/kg d.w. (mean ± SD, *n* = 10). Results for arsenic concentration in soluble extracts (mg/kg d.w., *n* = 1) are given in the rightmost column

Factors: coded (real)								
Experiment	A Sample weight (g)	B Type of extraction solution	C Volume of extraction solution (mL)	D Addition of H_2_O_2_	E = ABC Extraction temperature (°C)	F = BCD Extraction time (min)	G = ACD Use of ultrasonication	As conc. (mg/kg d.w.)
1	−1 (0.2)	−1 (water)	+1 (15)	−1 (No)	+1 (90)	+1 (60)	+1 (Yes)	11.1
2	−1 (0.2)	+1 (30 mM HNO_3_)	−1 (5)	+1 (Yes)	+1 (90)	−1 (30)	+1 (Yes)	10.4
3	−1 (0.2)	+1 (30 mM HNO_3_)	−1 (5)	−1 (No)	+1 (90)	+1 (60)	−1 (No)	10.4
4	+1 (0.5)	+1 (30 mM HNO_3_)	−1 (5)	−1 (No)	−1 (25)	+1 (60)	+1 (Yes)	10.1
5	+1 (0.5)	+1 (30 mM HNO_3_)	−1 (5)	+1 (Yes)	−1 (25)	−1 (30)	−1 (No)	9.9
6	−1 (0.2)	−1 (water)	−1 (5)	+1 (Yes)	−1 (25)	+1 (60)	+1 (Yes)	10.2
7	+1 (0.5)	−1 (water)	−1 (5)	+1 (Yes)	+1 (90)	+1 (60)	−1 (No)	10.6
8	+1 (0.5)	+1 (30 mM HNO_3_)	+1 (15)	+1 (Yes)	+1 (90)	+1 (60)	+1 (Yes)	10.5
9	−1 (0.2)	+1 (30 mM HNO_3_)	+1 (15)	+1 (Yes)	−1 (25)	+1 (60)	−1 (No)	10.0
10	+1 (0.5)	−1 (water)	−1 (5)	−1 (No)	+1 (90)	−1 (30)	+1 (Yes)	10.3
11	+1 (0.5)	−1 (water)	+1 (15)	+1 (Yes)	−1 (25)	−1 (30)	+1 (Yes)	10.3
12	−1 (0.2)	−1 (water)	−1 (5)	−1 (No)	−1 (25)	−1 (30)	−1 (No)	10.4
13	+1 (0.5)	+1 (30 mM HNO_3_)	+1 (15)	−1 (No)	+1 (90)	−1 (30)	−1 (No)	10.3
14	+1 (0.5)	−1 (water)	+1 (15)	−1 (No)	−1 (25)	+1 (60)	−1 (No)	10.1
15	−1 (0.2)	+1 (30 mM HNO_3_)	+1 (15)	−1 (No)	−1 (25)	−1 (30)	+1 (Yes)	10.3
16	−1 (0.2)	−1 (water)	+1 (15)	+1 (Yes)	+1 (90)	−1 (30)	−1 (No)	10.8

For the extraction, 0.2 g or 0.5 g of the blue mussel sample was weighed into 50-mL polypropylene tubes. Five or 15 mL of water or 30 mM HNO_3_ was added. Depending on the experimental set-up (Table 1), H_2_O_2_ was added to the extraction solution to yield a concentration of 1% H_2_O_2_ (v/v). A vortex mixer (IKA, Staufen, Germany) was used for 10 s, and then the tubes were placed in a water bath (OLS200, Grant, Cambridge, UK) at 25 °C or 90 °C, and left shaking (100 rpm) for 30 or 60 min. Selected tubes were ultrasonicated afterwards (Table 1). Subsequently, the tubes were placed in a centrifuge (1780×*g*, 10 min; Eppendorf Centrifuge 5702, Hamburg, Germany). The extracts (soluble fraction) were filtered using a 5-mL single-use syringe (Henke-Sass Wolf, Tuttlingen, Germany) connected to a 0.45-μm syringe filter (Sartorius, Göttingen, Germany) and transferred to new polypropylene tubes. The tubes with the residues (non-soluble fraction) were placed in a drying oven (60 °C, Fisher Scientific, Ottawa, Ontario, Canada) and left to dry for 2 days. Both soluble and non-soluble fractions were analyzed for total As using ICP-MS, while a portion of the soluble fraction was diluted with water (1:4, v/v) in a 1-mL polypropylene HPLC vial, and analyzed for As speciation using HPLC-ICP-MS.

### Optimization of factors with significant effects: extraction solution

To further optimize, extraction efficiencies of pure water and aqueous methanol (MeOH:H_2_O, 50% v/v) were compared using the blue mussel sample and CRMs (BCR 627, ERM-CD200, DORM-3, and TORT-3). Briefly, 0.2 g of sample was weighed into a 13-mL polypropylene tube. Five milliliters of pure water or aqueous methanol (MeOH:H_2_O, 50% v/v) was added, followed by vortex mixing. The tubes were placed in a shaking water bath (90 °C, 100 rpm) for 30 min and centrifuged (1780×*g*, 10 min). The soluble fraction was filtered using a 5-mL single-use syringe connected to a 0.45-μm syringe filter, transferred to new tubes, and analyzed for total As by ICP-MS and As speciation by HPLC-ICP-MS. Three replicates were performed for each sample.

### Total As determination by ICP-MS

Total As was determined by microwave digestion followed by analysis in ICP-MS, as described by Julshamn et al. [43]. Briefly, 0.2 g of sample was weighed into quartz tubes and added with 2 mL HNO_3_ and 0.5 mL H_2_O_2_. The tubes were capped and placed in a single-reaction-chamber microwave system (UltraWAVE, Milestone, Sorisole, Italy) for digestion. The digested solutions were allowed to cool then quantitively transferred to a 25-mL volumetric flask and diluted with water. The same digestion procedure was applied to the non-soluble and soluble fractions; only here, the sample weights were 0.2 g ± 0.1 g (mean ± standard deviation (SD), *n* = 16) for the non-soluble fraction (depending on how much residue was left) and 0.25 g for the soluble fraction. Total As analysis was carried out with an iCAP Q ICP-MS (Thermo Scientific, Waltham, MA, USA) equipped with an SC-4 DX autosampler (Elemental Scientific, Mainz, Germany). Daily instrument optimization was conducted following the manufacturer’s instructions. A complete list of instrument settings is given in Table 2. Instrument control and data processing were carried out through the Qtegra software (v. 2.10, 2018, Thermo Scientific, Waltham, MA, USA). For analyte quantification, calibration standard solutions were prepared by serially diluting appropriate amounts of a stock solution of As with aqueous 5% HNO_3_. The resulting calibration curve ranged from 0.5 to 25 μg/L. To compensate for possible instrumental drifts and matrix effects, online internal standard addition of germanium was employed. As part of quality control, TORT-3 and SRM 1566b were analyzed in duplicate in each analytical series and were used to evaluate method accuracy.

**Table 2:** The operating parameters for ICP-MS and HPLC-ICP-MS

Instrument settings		
ICP-MS settings	iCap Q	
RF power	1550 W	
Plasma gas flow	14.0 L/min	
Carrier gas flow	1.02 L/min	
Makeup gas flow	0.80 L/min	
Dwell time	0.1 s per isotope	
Isotopes monitored	^75^As, ^72^Ge (internal standard)	
HPLC-ICP-MS settings	1260 HPLC and 7900 ICP-MS	
RF power	1550 W	
Nebulizer gas flow	1.03 L/min	
Plasma gas flow	15.0 L/min	
Spray chamber temperature	2 C	
Isotopes monitored	^75^As, ^35^Cl	
Integration time	1 s	
	Cation-exchange	Anion-exchange
Guard column	Metrosep C 6 Guard (4.0 mm)	PRP-X100 Guard cartridge, PEEK
Analytical column	Metrosep C 6 (250 × 4.0 mm, 5 um)	PRP-X100 (250 × 4.6 mm, 5 um)
Mobile phase	A: 0 mM pyridine, 0.5% ACN, pH 2.7 B: 50 mM pyridine, 0.5% ACN, pH 2.7	A: 0.5 mM (NH_4_)_2_CO_3_, 3% MeOH, pH 9.3 B: 60 mM (NH_4_)_2_CO_3_, 3% MeOH, pH 9.3
Gradient	0–8 min (10% B), 8–10 min (10% to 100% B), 10–20 min (100% B), 20–23 min (10% B)	0–6 min (20% B), 6–17 min (100% B), 17–20 (20% B)
Flow rate	0.9 mL/min	1 mL/min
Injection volume	50 μL	50 μL

### As speciation by HPLC-ICP-MS

As speciation was achieved using cation- and anion-exchange methods using a 1260 Infinity HPLC coupled to a 7900 ICP-MS (Agilent Technologies, Santa Clara, CA, USA). The cation-exchange settings were based on previous studies [21, 23] and were further optimized in this work. A Metrosep C 6 column (250 × 4.0 mm, 5 μm, Metrohm, Herisau Switzerland), filled with silica gel with carboxyl groups, and a corresponding guard column were used to separate the cationic species. For the mobile phase, appropriate amounts of pyridine were diluted in aqueous 0.5% (v/v) acetonitrile to the desired ionic strength and subsequently adjusted to pH 2.7 with formic acid. The anion-exchange conditions were also developed based on previous works [21, 27]. A PRP-X100 column (250 × 4.6 mm, 5 μm, Hamilton, Reno, NV, USA), filled with polystyrene-divinylbenzene copolymer with quaternary ammonium group, and a corresponding guard column were utilized. The mobile phase was prepared by dissolving appropriate amounts of ammonium carbonate in aqueous 3% (v/v) methanol to the desired ionic strength and adjusted to pH 9.3 with ammonia. Mobile phases were vacuum-filtered through a 0.45-μm PTFE filter (Agilent Technologies, Santa Clara, CA, USA) prior to use. Gradient elution was implemented for both cation- and anion-exchange separations. The optimized HPLC-ICP-MS settings are also presented in Table 2.

For the quantification of analytes, mixed calibration standard solutions were prepared by serial dilution of appropriate amounts of stock solutions in aqueous methanol (MeOH:H_2_O, 50% v/v). External calibration curves were generated, and chromatographic peak areas were used for the quantification. Chromatographic peaks for the sample extracts were identified by comparison of retention time (RT) with the standards. Unknown peaks were quantified using the calibration curve of the As species with closest retention time. For quality control, CRMs were included in every analytical series. Extraction blanks were also analyzed to check for possible contamination. Instrument control and data processing were facilitated through the MassHunter 4.5 Workstation software (v. C.01.05, Agilent Technologies, Santa Clara, CA, USA).

### Statistical analysis and data treatment

For the fractional factorial design, statistical significance of the main effects was evaluated using analysis of variance (ANOVA) with a 95% confidence interval. In comparing the extraction efficiencies of pure water and aqueous methanol (MeOH:H_2_O, 50% v/v), a *t*-test was used to assess whether the results of the two extractants were significantly different. Statistica (v. 13.5.0.17, TIBCO, Palo Alto, CA, USA) was used in generating the experimental design and processing the corresponding analytical results. Microsoft Excel (Microsoft, Redmond, WA, USA) was used in statistical treatment of data and calculation of other analytical figures of merit. OriginPro 2020b (v. 9.7.5.184, OriginLab, Northampton, MA, USA) was used in creating figures.

## Results and discussion

### Total As in the pooled blue mussel sample and CRMs

The average total As concentration for the pooled blue mussel sample was 14.6 ± 0.1 mg/kg (mean ± SD, *n* = 10). This value was set as the target total As concentration and was used to calculate extraction efficiencies in the experimental design. Total As concentrations and extraction efficiencies for the different CRMs are given in Table 3. Based on *t*-test results, obtained total As concentrations were not significantly different from the certified values (95% confidence level).

**Table 3:** Arsenic concentrations in the CRMs and the blue mussel sample, soluble and non-soluble fractions, with calculated parameters for arsenic mass balance (mean ± SD, *n* = 5)

Arsenic species	BCR 627	CE278k	DORM-4	SQID-1	DOLT-5	TORT-3	CRM 7405-b	Blue mussel
Total As (mg/kg)	4.4 ± 0.1	6.7 ± 0.1	6.95 ± 0.08	16.4 ± 0.2	31.7 ± 1.0	64.7 ± 2.0	48.2 ± 1.0	14.6 ± 0.1
Certified value (mg/kg)	4.8 ± 0.3	6.7 ± 0.4	6.87 ± 0.44	14.1 ± 2.2	34.6 ± 2.4	59.5 ± 3.8	49.5 ± 1.0	
Soluble As (mg/kg)	4.3 ± 0.1	4.4 ± 0.1	5.68 ± 0.14	14.7 ± 1.3	32.6 ± 2.6	63.5 ± 0.8	27.3 ± 0.9	10.8 ± 0.6
Extraction efficiency (%)^^^	97 ± 1	66 ± 2	82 ± 2	90 ± 8	103 ± 8	98 ± 1	57 ± 2	74 ± 4
Sum of As species (mg/kg)^*^	4.3 ± 0.1	3.7 ± 0.1	5.49 ± 0.06	14.2 ± 0.3	29.6 ± 1.9	55.2 ± 1.1	27.6 ± 0.7	10.1 ± 0.2
Non-soluble As (mg/kg)	0.5 ± 0.1	2.9 ± 0.1	1.64 ± 0.07	3.5 ± 0.9	4.9 ± 0.4	8.4 ± 0.2	24.5 ± 1.2	4.3 ± 0.1
Sum As (mg/kg)^¤^	4.8 ± 0.1	7.3 ± 0.1	7.3 ± 0.2	18.2 ± 0.4	37.6 ± 2.9	71.9 ± 0.6	51.8 ± 0.4	15.1 ± 0.6
As recovery (%)^¤¤^	109 ± 2	109 ± 2	105 ± 3	111 ± 3	118 ± 9	111 ± 1	107 ± 1	104 ± 4

^^^Extraction efficiency = (Soluble As/Total As) × 100

^*^Sum of As species = Sum of chromatographed peaks

^¤^Sum As = Soluble As + Non-soluble As

^¤¤^As recovery = (Sum As/Total As) × 100

### Extraction optimization: screening of factors using fractional factorial design

**Fig. 2:**
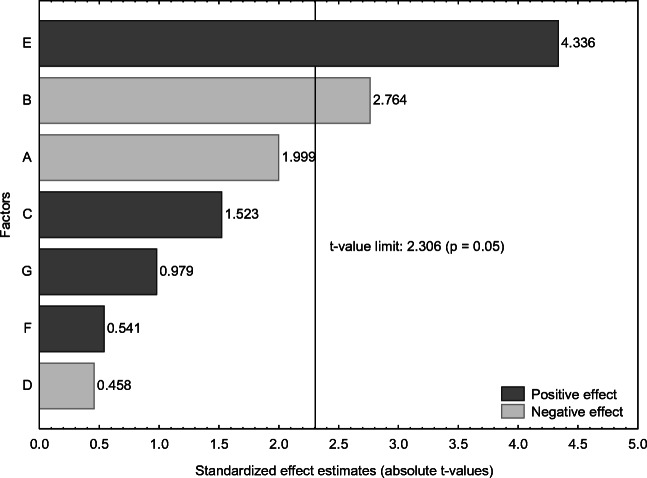
Pareto chart of standardized effects with soluble As concentration as the response. The factors investigated were (A) sample weight (g), (B) type of extraction solution, (C) volume of extraction solution (mL), (D) addition of H_2_O_2_ in the extraction solution, (E) extraction temperature (°C), (F) extraction time (min), and (G) use of ultrasonication. The *t*-value limit was 2.306 (*p* = 0.05), above which signifies statistical significance. Bars in dark gray and light gray represent positive and negative effects, respectively

As shown in Table 1, the soluble As concentration from the experiments ranged from 9.9 to 11.1 mg/kg, with experiments 5 and 1 posting the lowest and highest recoveries, respectively. These correspond to 68% to 76% of the total As concentration of the blue mussel sample (14.6 mg/kg). Figure 2 shows the Pareto chart of standardized effect estimates of the different factors. The critical *t*-value denoting statistical significance was 2.306 (*p* = 0.05). Factors with *t*-values above this limit have significant effects on the response (soluble As concentration). The significant factors were extraction temperature (E) and type of extraction solution (B), having positive and negative effects, respectively (Fig. 2). This suggests that extraction temperature should be kept at the “+1” setting, while the extraction solution at the “−1” setting. As shown in Table 1, the effect of extraction temperature is aliased by a three-factor interaction (i.e., E = ABC), which is a caveat of using fractional factorial design. However, by choosing a 2^7–3^ fractional factorial design, resolution IV was achieved. Main effects are only aliased with three-factor interactions and higher, which are often non-significant. This approach reduces the likelihood of making false interpretations [33].

### Optimization of significant factors

When using multivariate techniques during method development, screening experiments are usually followed by further optimization using response surface methodology (RSM). The use of RSM models the relationship between the factors and the response/s, and identifies factor settings which will give the maximum (or minimum) response [33]. In the present work, the significant factors determined were extraction temperature and type of extraction solution. The high-level setting of extraction temperature in the screening experiments was already at 90 °C. Similar studies have explored applying up to 85 °C only for the extraction of arsenic [34, 35]. If boiling or higher temperatures are required, an oil bath would be more appropriate to use. Thus, due to equipment limitation and safety consideration, the extraction temperature was fixed at 90 °C.

With only one factor left to optimize, a univariate approach was implemented instead of RSM. Furthermore, the type of extraction solution is a non-numerical, discontinuous variable, so the use of RSM, which generates polynomials based on quantitative variables [32, 33], is not entirely applicable. From Fig. 2, low-level setting (pure water) was preferred for the extractant. Hence, other aqueous-based extraction solutions were considered. In this study, the extraction efficiencies of aqueous methanol (MeOH:H_2_O, 50% v/v) and pure water were compared. As confirmed by *t*-test results, extraction efficiencies were significantly higher for BCR-627, DORM-3, and the blue mussel sample when using aqueous methanol (MeOH:H_2_O, 50% v/v) (see Supplementary Information (ESM) Fig. S1). The highest increase was seen for DORM-3 with a 20% improvement. Most arsenic species in marine samples are water-soluble; however, the addition of methanol increases the solubility of less polar arsenic species which are not extracted with water [44]. In contrast, there was a non-significant difference observed for extraction efficiencies for ERM-CD200 (seaweed) and TORT-3 (lobster hepatopancreas) (Fig. S1). An overall high extraction recovery was observed when using aqueous methanol (MeOH:H_2_O, 50% v/v), with over 90% of total As extracted in most samples. The only exceptions were the blue mussel and seaweed CRM, having approximately 80% of the total As extracted. The non-extracted arsenic species are possibly lipid-soluble species and would require a different extraction strategy using more non-polar extraction solutions.

The applicability of MeOH:H_2_O solutions in extracting water-soluble As species has been widely documented [23, 24, 29]. Aqueous methanol has also been used in extracting arsenolipids, although a higher percentage of methanol is often applied (e.g., MeOH:H_2_O, 9:1 v/v) [45, 46]. In this regard, the use of methanol might co-extract polar arsenolipids causing an apparent increase in extraction efficiency but will subsequently be unquantified since they will elute with the void volume. To verify if this is the case, the chromatographic recoveries were checked to ensure that extracted As species are accounted for. The chromatographic recoveries obtained were between 84 and 103% (Table 3), suggesting that the extracted arsenic species were sufficiently quantified with the proposed method.

From the results of the screening and optimization experiments, the optimum extraction conditions were identified: 0.2-g sample weight, 5 mL of aqueous methanol (MeOH:H_2_O, 50% v/v) as extraction solvent, extraction temperature of 90 °C, and extraction time of 30 min. The non-significant factors were kept at low levels in line with “Green Chemistry” principles [47].

### Optimization of HPLC-ICP-MS parameters

#### Column selection

Water-soluble As compounds have different pK_a_ values which lead to formation of anionic or cationic species in aqueous solutions depending on the pH. Hence, a single chromatographic approach is usually not feasible, and the combined use of cation- and anion-exchange chromatography is consequently recommended [21, 26, 41, 45]. For cation-exchange, columns which were typically used in previous studies include IonoSpher 5C [23, 45], Zorbax 300 SCX [26], and Metrosep C 6 [21]. In the present work, IonoSpher 5C and Metrosep C 6 were explored since they have been reported to separate the largest number of cationic species [21, 23]. However, a shift in RT was observed for TMAO when IonoSpher 5C was used in between days. Similar poor reproducibility when using IonoSpher columns has previously been reported [23, 44]. The findings were attributed to both chemical properties of the compounds and endogenous matrix components. In line with these observations, Metrosep C 6 was chosen as the cation-exchange column for succeeding experiments. For anion-exchange, PRP-X100 was applied in the present work, which has been the most commonly used column for As speciation analysis in marine matrices [1].

#### Buffer selection and effect of pH

For the mobile phase, cationic As species are normally eluted by pyridine-based solutions [21, 23, 26]. For anionic As species, phosphate- [48], carbonate- [49], and nitrate-based eluents [25, 41] are utilized. In this work, ammonium carbonate was used as the mobile phase buffer for anion-exchange and pyridine for cation-exchange.

**Fig. 3:**
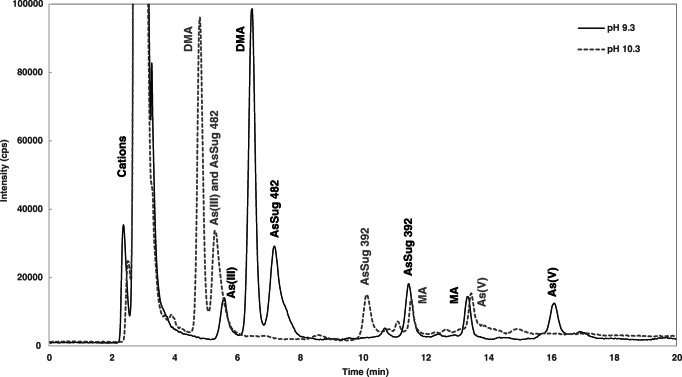
Overlaid chromatograms of anion-exchange separation of TORT-3 using mobile phase with different pH 9.3 (solid line) and pH 10.3 (dotted line)

Ion-exchange chromatography relies on electrostatic interactions between functional groups of the stationary phase and the charged analytes, as influenced by the mobile phase pH and pK_a_ of the As compounds [20]. To evaluate the effect of pH on the retention of analytes, two pH values were tested for anionic separation using ammonium carbonate as buffer: 9.3 and 10.3. It was seen that analytes were more retained at pH 9.3, as shown in the comparison of two chromatograms of TORT-3 in Fig. 3. At this pH, carbonate ions exist primarily as HCO_3_^−^, whereas at pH 10.3, carbonic acid has reached its second dissociation equilibrium, causing an increase of CO_3_^2−^ ions. Since CO_3_^2−^ ions have stronger affinity to the quaternary ammonium groups in the stationary phase, anionic species were eluted more easily. It was also noted that the chromatographic peak for As(III) disappeared at pH 10.3 while the peak area for AsSug 482 slightly increased, suggesting a shift in RT for As(III). This was confirmed by a spiking experiment with As(III) to a TORT-3 extract (data not shown). This shift in RT can be explained by the first pK_a_ of As(III) being 9.23 [50]; hence, at pH 10.3, the dominant form is the deprotonated H_2_AsO_3_^−^. The increase of negatively charged ions results in a stronger interaction with the stationary phase; thus, As(III) is more retained and elutes in the RT of AsSug 482 (Fig. 3). Other As species were not affected since their pK_a_ values are much lower. Due to the foregoing observations, pH 9.3 was identified as the optimum pH for a carbonate-based mobile phase. For the cationic separation, pH 2.7 was chosen for a pyridine-based eluent since this has been demonstrated to work well in previous studies [21, 23], and was also confirmed in the present study.

### Carbon-induced signal enhancement

The effect of adding organic solvent to the mobile phase to improve ICP-MS sensitivity has been extensively described in the literature [51–53]. An increase in signal is desirable, particularly for As which has a high ionization potential and consequently not quantitatively ionized in the argon plasma of the ICP-MS [51]. Thus, the effect of addition of methanol and acetonitrile concentrations to the mobile phases was optimized in the present study. It has been stated that methanol, or alcohols, in general, should not be used with cation-exchange columns with carboxyl groups due to possible esterification of ion-exchange sites [54]. Hence, ACN was chosen as the added organic solvent for cation-exchange chromatography using Metrosep C 6. Two sets of 5 μg/L standard solutions of As(V) containing different fractions of organic solvent were aspirated into the ICP-MS. Highest signal enhancement was achieved at 0.5% (v/v) ACN and 3% (v/v) MeOH, with four- and fivefold increase, respectively (ESM Fig. S2). The nitrogen atom in ACN may be contributing to the signal enhancement, similar to the increased signal intensity brought about by nitrogen gas in laser ablation ICP-MS [55]. In addition, MeOH is more volatile than ACN and, hence, would require less energy from the ICP for decomposition [53]. This could possibly explain why the ICP can tolerate a higher proportion of MeOH. The identified optimum MeOH concentration of 3% (v/v) is in accordance with the findings of Larsen et al. [51]. At concentrations beyond 0.5% (v/v) ACN and 3% (v/v) MeOH, the magnitude of signal enhancement started to decline. In fact, at ACN > 3.5% (v/v), the obtained intensity was even less than that without added ACN, suggesting signal suppression. The decline in intensity after reaching a certain threshold for organic solvent is commonly attributed to the cooling effect on the plasma, which decreases the plasma temperature and hampers the efficient ionization of analytes [52, 53].

**Fig. 4:**
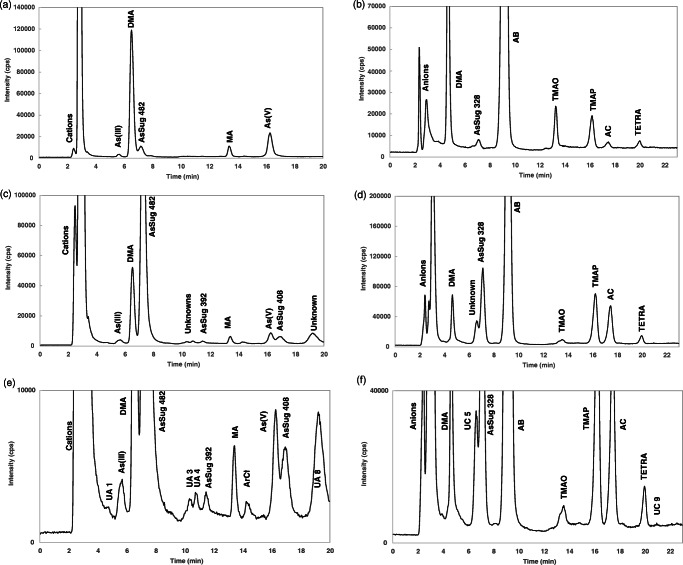
Chromatograms of arsenic species in a DORM-4 extract using (a) anion- and (b) cation-exchange HPLC-ICP-MS. Chromatograms of arsenic species in a blue mussel extract using (c) anion- and (d) cation-exchange HPLC-ICP-MS. Enlarged chromatograms of (e) panel c and (f) panel d

Based on the experimental results, the optimum conditions for the pyridine-based mobile phase are pH 2.7 and 0.5% (v/v) ACN. For the carbonate-based eluent, pH 9.3 and 3% (v/v) MeOH were chosen. The optimized mobile phase compositions, together with the HPLC-ICP-MS settings (Table 2), allowed chromatographic separation of several As species, with peaks of sufficient intensity, and run time of less than 25 min. Sample chromatograms for DORM-4 and blue mussel are presented in Fig. 4. Chromatograms for the standard solutions can be found in the ESM (Fig. S3).

### Method validation

To demonstrate the applicability of the developed method, a single-laboratory validation was carried out according to Eurachem’s recommendations [56]. Due to limited availability of standards, some method validation parameters (i.e., working range, linearity, spiking recovery, and precision) could not be calculated for all methylated arsenic species and arsenosugars (i.e., DMAA, DMAP, AsSug 328, AsSug 392, AsSug 408, and AsSug 482).

In this study, spectral interference of ^40^Ar^35^Cl^+^ with As (*m*/*z* 75) was avoided by employing a gradient profile which chromatographically separated the chloride from the rest of the anionic As species. The retention time for chloride under anion-exchange settings was 14.6 min, while the closest eluting analytes were MA (13.4 min) and As(V) (16.3 min), hence, no coelution of chloride with the As species.

The limit of detection (LOD) was calculated as three times the SD of ten replicates of a 0.5 μg/L mixed standard solution subjected to the extraction procedure and analyzed with HPLC-ICP-MS, while the limit of quantification (LOQ) was set as ten times the SD. The LOQ values ranged from 0.005 to 0.025 mg/kg for the different species (ESM Table S1). The obtained LOD and LOQ values were comparable with those reported elsewhere [21, 26, 27].

Linearity was assessed by analyzing in triplicate a blank and six different concentration levels of As standard solutions. The response (peak area) was plotted against concentration and appropriate regression statistics were calculated. Obtained correlation coefficients (*r*) were at least 0.999 (ESM Table S1). Statistical analysis of residuals also demonstrated random distribution, hence, confirming good linearity of the method. The concentration levels used for the linearity experiments also represent the method working range (ESM Table S1).

Trueness was evaluated in two ways: (i) analysis of CRMs and (ii) analysis of spiked samples. As shown in Table 4, good agreement was found for the experimental results compared with certified and information values, with recoveries in the range of 88 to 109% of the certified concentrations. In addition, BCR 627, DORM-4, and the blue mussel sample were spiked at three concentration levels (0.3, 0.5, and 1 mg/kg for AB and DMA; 0.1, 0.3, and 0.5 mg/kg for others) in duplicate. The average spiking recoveries for the three sample types were in the range of 83 to 120%, demonstrating that the integrity of species has been maintained throughout the analytical procedure. Wolle et al. [21] reported poor recoveries (<50%) for TMAO, DMAA, DMAP, DMAE, and As(III) in (non-freeze dried) cod, haddock, and shrimp which were attributed to the binding and interconversion of species due to endogenous matrix components. The problem was resolved with the addition of N-ethylmaleimide.

**Table 4:** Concentrations of arsenic species in the CRMs and the blue mussel sample using the validated method, alongside certified and information values for comparison (mean ± SD, *n* = 5)

Arsenic species	BCR 627	CE278k	DORM-4	SQID-1	DOLT-5	TORT-3	CRM 7405-b	Blue mussel sample
Anions								
As (III)	<0.025	0.064 ± 0.002	<0.025	<0.025 (0.019)	0.125 ± 0.008	0.361 ± 0.012	0.429 ± 0.006	0.043 ± 0.004
DMA	0.155 ± 0.004 **(0.15 ± 0.02)**	0.636 ± 0.013	0.618 ± 0.006	0.032 ± 0.003 (0.03)	1.870 ± 0.120	1.181 ± 0.030	0.286 ± 0.005 (0.24)	0.266 ± 0.006
DMAA	<0.017	0.162 ± 0.007	0.055 ± 0.004	0.055 ± 0.006	0.166 ± 0.009	0.278 ± 0.028	-	0.091 ± 0.005
AsSug 482	0.041 ± 0.002	0.244 ± 0.007	0.068 ± 0.001	0.026 ± 0.003	0.234 ± 0.017	0.545 ± 0.013	0.313 ± 0.004 (0.20)	1.329 ± 0.052
AsSug 392	-	-	-	-	-	0.195 ± 0.019	0.178 ± 0.010 (0.16)	<0.011
MA	<0.011	0.039 ± 0.001	0.046 ± 0.003	<0.011	0.100 ± 0.007	0.131 ± 0.011	0.080 ± 0.005	0.024 ± 0.001
As (V)	0.035 ± 0.001	0.037 ± 0.010	0.110 ± 0.004	0.032 ± 0.006 (0.028)	0.093 ± 0.004	0.270 ± 0.024	24.3 ± 0.6 **(24.4 ± 0.7)**	0.032 ± 0.005
AsSug 408	-	-	-	-	-	0.195 ± 0.035	1.36 ± 0.03 **(1.41 ± 0.04)**	0.028 ± 0.006
Sum of unknown anions	–	0.024 ± 0.001 (2 unknowns)	0.007 ± 0.001 (1 unknown)	-	0.071 ± 0.004 (2 unknowns)	0.105 ± 0.002 (3 unknowns)	**-**	0.101 ± 0.001 (4 unknowns)
Cations								
AsSug 328	0.008 ± 0.001	0.087 ± 0.003	0.027 ± 0.002	0.020 ± 0.002	0.118 ± 0.009	2.315 ± 0.064	0.415 ± 0.002 **(0.44 ± 0.02)**	0.689 ± 0.015
DMAP	-	<0.007	-	-	-	-	0.013 ± 0.001	<0.007
AB	3.94 ± 0.09 **(3.9 ± 0.2)**	2.24 ± 0.04	4.32 ± 0.05 **(3.95 ± 0.36)**	13.6 ± 0.3 **(13.96 ± 0.54)**	26.1 ± 1.7 **(24.2 ± 0.8)**	48.5 ± 1.0 **(54.9 ± 2.5)**	-	6.46 ± 0.08
TMAO	<0.012	<0.012	0.091 ± 0.003	0.020 ± 0.003	0.156 ± 0.017	0.161 ± 0.006	-	0.044 ± 0.001
TMAP	0.023 ± 0.001	0.089 ± 0.002	0.068 ± 0.001	0.347 ± 0.032	0.338 ± 0.024	0.308 ± 0.008	-	0.323 ± 0.004
AC	0.016 ± 0.002	<0.007	0.017 ± 0.001	0.033 ± 0.003	0.115 ± 0.009	0.037 ± 0.002	-	0.369 ± 0.005
TETRA	0.033 ± 0.001	0.028 ± 0.001	<0.018	<0.018	0.086 ± 0.012	0.138 ± 0.003	-	0.057 ± 0.001
Sum of unknown cations	0.022 ± 0.001 (1 unknown)	0.017 ± 0.001 (4 unknowns)	0.016 ± 0.001 (3 unknowns)	0.017 ± 0.001 (3 unknowns)	0.069 ± 0.004 (2 unknowns)	0.455 ± 0.009 (4 unknowns)	0.208 ± 0.004 (2 unknowns)	0.198 ± 0.002 (4 unknowns)
Sum of As species (mg/kg)	4.3 ± 0.1	3.7 ± 0.1	5.49 ± 0.06	14.2 ± 0.3	29.6 ± 1.9	55.2 ± 1.1	27.6 ± 0.7	10.1 ± 0.2
Soluble As (mg/kg)	4.3 ± 0.1	4.4 ± 0.1	5.68 ± 0.14	14.7 ± 1.3	32.6 ± 2.6	63.5 ± 0.8	27.3 ± 0.9	10.8 ± 0.6
Chromatographic recovery (%)	100 ± 3	84 ± 1	96 ± 1	97 ± 9	88 ± 4	88 ± 2	103 ± 4	94 ± 5

Bolded numbers in parenthesis are certified values. Underlined numbers in parenthesis are information values

Chromatographic recovery = (Sum of As species/Soluble As) × 100

DMAE was not detected in any of the samples analyzed

“-”, not detected

Precision was evaluated in terms of repeatability by performing five replicate analyses for the blue mussel sample and CRMs. The calculated RSD values for the obtained As species concentrations ranged from 1 to 28%. Concentrations close to LOQ registered the highest RSDs. Average RSDs for the spiked concentration levels were also calculated and were in the range of 0.1 to 10.7% for BCR 627, DORM-4, and the blue mussel sample. The general trend was that higher spiking concentrations yielded better precision.

Measurement uncertainty was estimated using the simplified approach proposed by Barwick et al. [57], wherein results from trueness and precision studies were used to calculate the standard uncertainty. The expanded uncertainty was obtained by multiplying the standard uncertainty by a coverage factor (*k* = 2; 95% confidence interval). Calculated expanded uncertainties were in the range of 2 to 67%, where the highest expanded uncertainties were associated with analytes in concentrations close to LOQ.

### Arsenic species in certified reference materials

Good chromatographic recoveries (84 to 103%) were achieved for all CRMs and the blue mussel sample when using the speciation method developed. The obtained concentrations for the As species in the CRMs and the blue mussel sample, together with certified and information values, are shown in Table 4. Due to its presence in the market for more than 20 years, BCR-627 is one of the most utilized CRMs in As speciation. In this study, the obtained AB and DMA concentrations were 3.94 ± 0.09 mg/kg and 0.155 ± 0.004 mg/kg, respectively. These are in accordance with the certified values for AB and DMA of 3.9 ± 0.2 mg/kg and 0.15 ± 0.02, respectively. For BCR 627, literature values for AB generally range from 3.6 to 3.9 mg/kg, while DMA results normally vary from 0.13 to 0.15 mg/kg. For DORM-4, the concentrations found for AB and DMA were 4.32 ± 0.05 mg/kg and 0.618 ± 0.006 mg/kg, respectively. These agree with the certified value for AB which is 3.95 ± 0.36 mg/kg, and with literature values ranging from 3.74 to 4.02 mg/kg for AB, and 0.54 to 0.94 mg/kg for DMA [21, 58–60]. Other CRMs were just recently introduced; hence, limited amount of data is available. To date, Wolle et al. [21] have reported the most extensive work by quantifying as many as 35 known and unknown As species in two CRMs and a range of seafood samples. The present work aims to augment this effort by reporting the concentrations of different As species in recent versions of the CRMs.

**Fig. 5:**
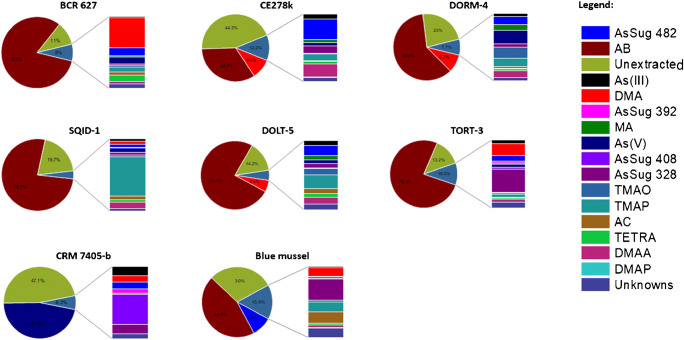
Arsenic species profile in the CRMs and the blue mussel sample analyzed. Arsenic species fraction, % = (concentration of As species/total As) × 100

As shown in Fig. 5, with the exception of CRM 7405-b, AB was the predominant As species in the majority of the tested CRMs, contributing as much as 77% of the total As. While arsenobetaine is mainly found in fish, it can also exist as major As species in e.g. crab and clam samples, and in minor proportions in shrimp [2]. DMA was also a major As species, although accounting for less than 10% of the total As. Other organoarsenic species such as MA, AC, TMAO, TMAP, AC, and TETRA were present in minor amounts (< 5%). In contrast, TETRA exists as a major species in mollusks [11]. Arsenosugars were quantified in all samples, but they were notable especially in the blue mussel sample, ERM CE278k (also a blue mussel), TORT-3, and CRM 7405-b (hijiki). Arsenosugars are not exclusively found in macroalgae, as they also appear in higher concentrations in clams, mollusks, and oyster tissue, and in trace levels in kelp [11]. The highest concentration of an arsenosugar was found in TORT-3 with 2.32 ± 0.06 mg/kg (AsSug 328), which is comparable to available literature data of 2.71 mg/kg [21]. Aside from arsenosugars, marine macroalgae are also known to contain elevated levels of iAs. In the present study, an As(V) concentration of 24.3 ± 0.6 mg/kg was found in CRM 7405-b, which is in accordance with the certified value (Table 4). Our results, as supplemented by available literature data, confirm that As exists in several forms and in various concentrations in a broad variety of marine matrices. Trace levels of As species can be found in matrices where one As form is predominant, but this does not imply cross-contamination. In addition, blank samples were regularly included throughout the analytical run and no “memory effects” were observed.

In the present work, most number of As peaks were detected in the blue mussel samples, with a total of 23 As peaks, where 8 peaks are unknown. A total of 33 peaks, 17 unknown (ESM Table S2) and 16 known arsenic species, were detected in the CRMs and the blue mussel sample analyzed. It should be clarified, however, that coelution with our approach cannot be completely ruled out, and further optimization of the chromatography may reveal additional unknown peaks.

## Conclusions

In this work, an extraction procedure for water-soluble As species in marine samples was optimized using a 2^7–3^ fractional factorial design. Extraction temperature and the type of extraction solution were identified as factors with significant effects. Based on recoveries for total As content, the optimum conditions were 0.2-g sample weight, 5 mL of aqueous methanol (MeOH:H_2_O, 50% v/v) as extractant, and extraction carried out at 90 °C for 30 min. Together with the optimized anion- and cation-exchange HPLC-ICP-MS parameters, these conditions allowed for satisfactory quantification of As species with low solvent and energy consumption. A single-laboratory validation was performed to demonstrate the applicability of the developed method. Different marine CRMs were used as test samples and satisfactory method performance characteristics were achieved. With a total of 33 known and unknown water-soluble species quantified, this study produced a new set of As speciation data which serves as indicator values for succeeding speciation studies.

## Supplementary information

ESM 1 (PDF 446 kb)
