# Supplementary material for: Speciation analysis of organoarsenic species in marine samples: method optimization using fractional factorial design and method validation
Source: Anal Bioanal Chem. 2021 May 15;413(15):3909–23. doi: 10.1007/s00216-021-03341-4 (PMC8189937; doi:10.1007/s00216-021-03341-4)
Supplement: Supplementary file 1 — (PDF 446 kb) [file 216_2021_3341_MOESM1_ESM.pdf]

Supplementary Information

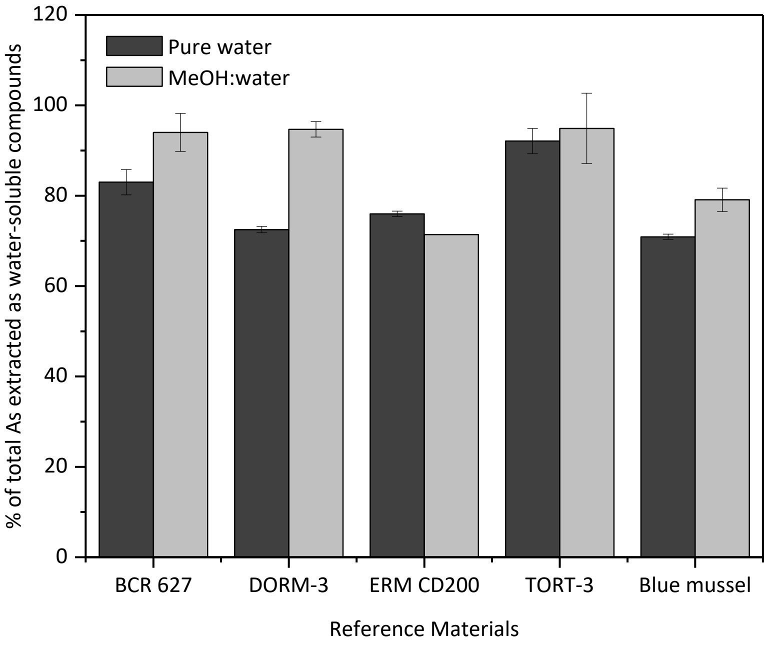

Fig. S1 Extraction efficiencies (EE) using pure water and aqueous methanol (MeOH:H_2O, 50% v/v) as extractant in the blue mussel sample and CRMs (mean ± SD, *n* = 3); EE = (total As in extract/total As in sample) x 100

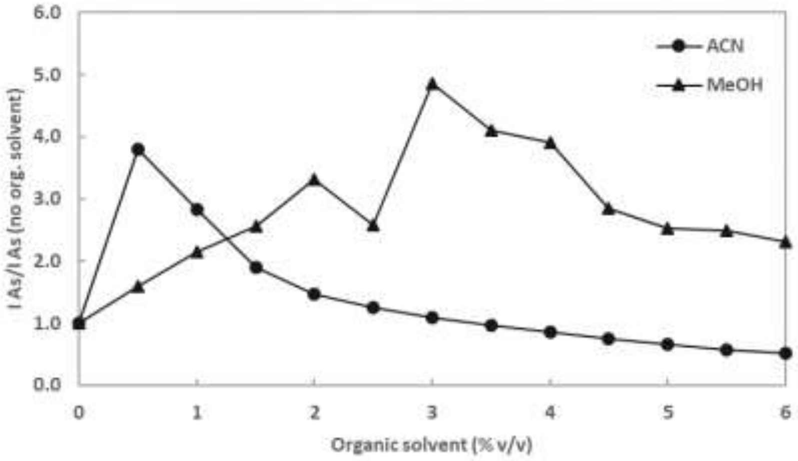

- **I As** – intensity of ^{75}As for a std solution with a certain % of organic solvent
- **I As (no org. solvent)** – intensity of ^{75}As for a std solution without org solvent

Fig. S2 Relative ICP-MS response for a 5 µg/L standard solution of As(V) in different proportions (%) of acetonitrile and methanol

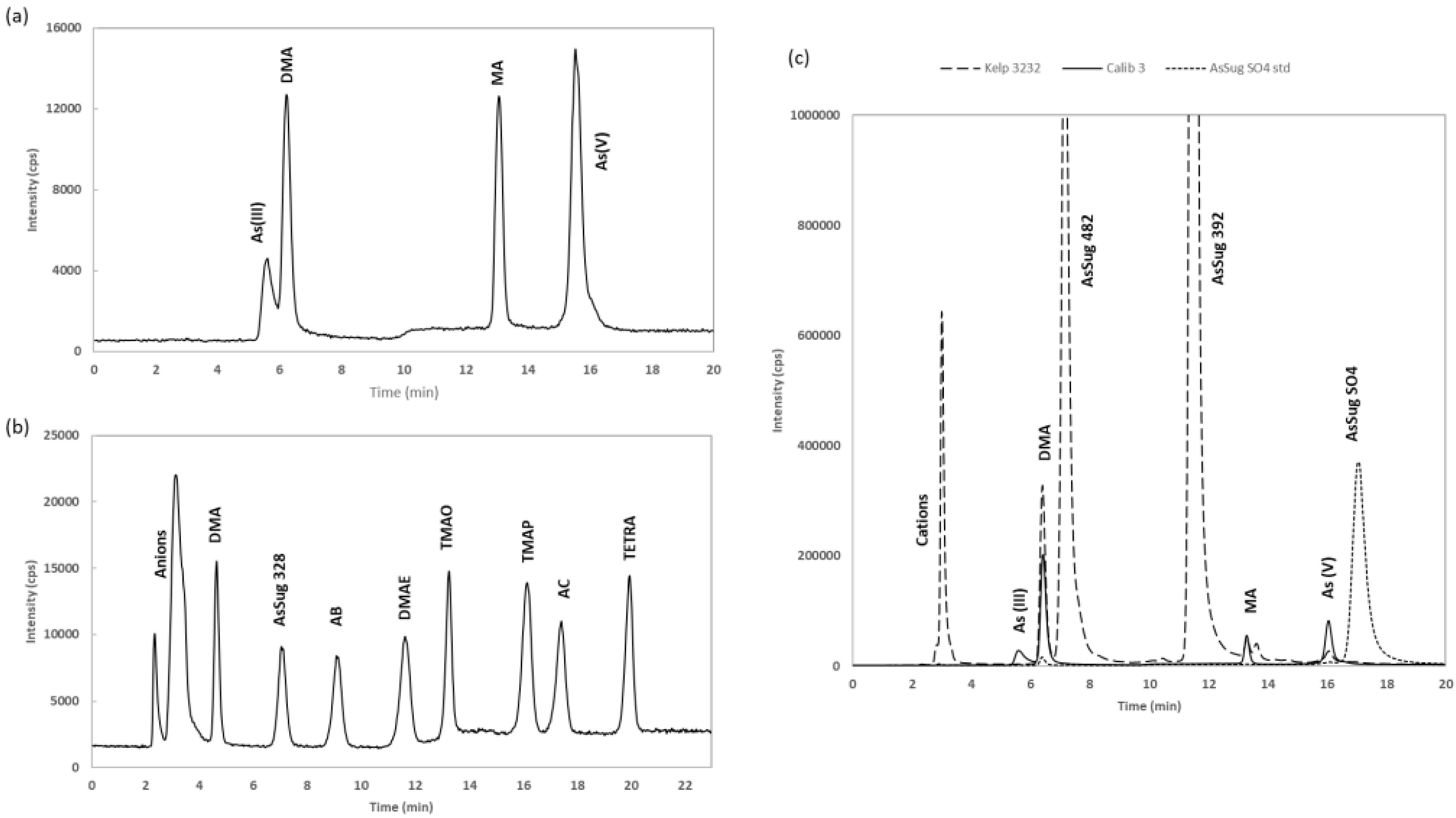

Fig. S3 Chromatograms of arsenic species in mixed standard solutions (~0.5 µg/L) using (a) anion- and (b) cation-exchange HPLC-ICP-MS. Due to limited/unavailable standard solutions of arsenosugars, overlaid anion-exchange chromatograms of SRM 3232 Kelp powder, an anion calibration standard, and a standard solution of AsSug SO4 are also provided to demonstrate retention times (c)

Table S1 LOD and LOQ values, alongside the working ranges and correlation coefficients (r) for the different arsenic species

Species	LOD (mg/kg)	LOQ (mg/kg)	Working range (µg/L)	r
As(III)	0.008	0.025	0.3-4.8	1.0000
DMA	0.005	0.017	1.0-15.7	0.9996
DMAA	0.005	0.017		
AsSug 482	0.005	0.017		
AsSug 392	0.003	0.011		
MA	0.003	0.011	0.3-5.3	0.9996
As(V)	0.004	0.013	0.3-4.8	0.9991
AsSug 408	0.004	0.013		
AsSug 328	0.002	0.007		
DMAP	0.002	0.007		
AB	0.003	0.010	18.9-66.1	0.9992
DMAE	0.006	0.019	0.6-5.7	1.0000
TMAO	0.004	0.012	0.4-4.4	0.9998
TMAP	0.001	0.005	0.5-5.5	0.9999
AC	0.002	0.007	0.5-4.5	0.9999
TETRA	0.005	0.018	0.5-4.5	0.9999

Table S2 Approximate concentrations of unknown arsenic species in the CRMs and the blue mussel sample using the validated method (mean ± SD, *n* = 5)

Species	RT (min)	BCR 627	CE278k	DORM-4	SQID-1	DOLT-5	TORT-3	CRM 7405-b	Blue mussel
Anions									
UA 1	4.5	-	-	-	-	-	0.055 ± 0.002	-	0.004 ± 0.001
UA 2	7.8	-	-	0.007 ± 0.001	-	-	-	-	-
UA 3	10.4	-	-	-	-	-	-	-	0.004 ± 0.001
UA 4	10.7	-	-	-	-	-	0.036 ± 0.003	-	0.006 ± 0.001
UA 5	12.5	-	-	-	-	-	0.014 ± 0.001	-	-
UA 6	15.4	-	-	-	-	0.032 ± 0.001	-	-	-
UA 7	18.0	-	0.009 ± 0.001	-	-	0.039 ± 0.007	-	-	-
UA 8	19.3	-	0.015 ± 0.002	-	-	-	-	-	0.087 ± 0.004
Cations									
UC 1	3.9	-	0.007 ± 0.001	0.007 ± 0.001	0.005 ± 0.001	0.035 ± 0.002	-	0.026 ± 0.003	0.005 ± 0.001
UC 2	4.4	-	-	-	-	-	0.169 ± 0.023	-	-
UC 3	5.2	-	-	-	-	-	0.149 ± 0.006	-	-
UC 4	5.3	-	-	-	-	-	-	0.182 ± 0.004	-
UC 5	6.6	0.022 ± 0.001	-	0.005 ± 0.001	0.004 ± 0.001	-	-	-	0.185 ± 0.005
UC 6	12.6	-	0.003 ± 0.001	0.004 ± 0.001	-	-	0.086 ± 0.004	-	-
UC 7	14.0	-	0.002 ± 0.001	-	-	-	-	-	-
UC 8	14.8	-	0.005 ± 0.001	-	-	-	-	-	0.004 ± 0.001
UC 9	21.0	-	-	-	0.007 ± 0.001	0.034 ± 0.006	0.051 ± 0.004	-	0.004 ± 0.001

UA: unknown anion
UC: unknown cation
'-': not detected
